# Attention Deficit Disorder and Allergic Rhinitis: Are They Related?

**DOI:** 10.1155/2016/1596828

**Published:** 2016-10-31

**Authors:** Isaac Melamed, Melinda Heffron

**Affiliations:** IMMUNOe Research Centers, Centennial, CO 80112, USA

## Abstract

The association between ADHD and allergy remains controversial. Our previous findings suggest that nerve growth factor may link the nervous and immune systems. The primary objective of this study was to determine if a combination of cetirizine + methylphenidate is effective in children with comorbid ADHD and allergic rhinitis. We also examined the role of nerve growth factor in these comorbidities. Our randomized, double-blind, placebo-controlled, crossover study enrolled 38 children diagnosed with comorbid ADHD and allergy using cetirizine (*n* = 12), sustained-release methylphenidate (*n* = 12), or cetirizine + methylphenidate (*n* = 14). Endpoints compared baseline to posttreatment evaluations for allergic rhinitis and ADHD scores. Serum nerve growth factor levels were measured using ELISA. For allergy endpoints, combination therapy produced results superior to individual therapy. For ADHD, similar scores were achieved for individual therapy; however, combination therapy resulted in improved scores. Nerve growth factor levels were downregulated following this trend. We conclude that ADHD and allergic rhinitis may have common mechanism and represent a comorbid condition that links the nervous system to the immune system. Further studies are needed.

## 1. Introduction

Allergic rhinitis affects up to 30% of all children. Some medications currently being used to treat this disorder, which significantly impacts learning, school performance, and attendance [1–3], may adversely affect the central nervous system and contribute to learning impairment [4]. In many cases, children with allergic rhinitis are also diagnosed with, and treated for, attention deficit/hyperactivity disorder (ADHD).

Attention disorders are among the most common chronic childhood and adolescent behavioral problems [5]. While the etiology of ADHD is unknown, it is most likely the result of interplay between various neuroanatomical/neurochemical systems and genetic and psychological factors [6]. An association between ADHD and allergy has long been touted but remains controversial [7–10]. It has also been suggested that children with ADHD and those with allergic disease may share a common biological background [7, 11, 12].

The role the immune system plays in allergic inflammation has been carefully examined [13]. Previously, we suggested that neurotrophic protein nerve growth factor (NGF) might serve as a vehicle for cross talk between the immune and nervous systems. We found that NGF (1) regulates immunoglobulin (Ig) production, particularly IgE regulation, and (2) plays a major role in the control of allergic inflammatory response, serving as one of the critical proteins in the cross talk between the nervous and immune systems and to the endocrine system [14–19]. We also demonstrated (1) the presence of functional NGF receptors on human B-lymphocytes and (2) that gp140trk-cells were the main active tyrosine kinase involved in NGF signaling [14].

Keeping in mind that ADHD is a complex, multifaceted illness and that it is critical that we understand the role allergic response plays in the etiology of the subset of children with both diseases, we devised our study to (1) promote an understanding of the synergistic effects of treatment with both antihistamines and stimulants, (2) provide insight into the pathophysiology of this illness, and (3) determine the role NGF may play in children with both ADHD and allergic rhinitis.

## 2. Methods

Ours was a randomized, 3-treatment group, double-blind, placebo-controlled, crossover study of 38 children with allergic rhinitis and ADHD. Approved by the local IRB, it was conducted in Colorado over a 4-year period during the tree and grass allergy seasons. We chose this period of time in order to capture results during the allergy season over an extended period of time. Subject inclusion criteria included boys or girls aged 8 to 18 years with a diagnosis and history of seasonal allergic rhinitis to a prevalent allergen (documented by a recognized skin test, skin prick, or intradermal [ID]), which required pharmacologic treatment at the time of enrollment and for the previous 2 years (prick wheal >3 mm over negative control; intradermal wheal >5 mm over negative control) and a diagnosis of ADHD and treatment regimen with stimulant medication.

After obtaining written informed consent from a parent, legal guardian, or subjects of legal age, participants were randomized and screened using the Multidimensional Assessment of Anxiety in Children (MASC) [20]; the Children's Depression Inventory (CDI) [21]; and the Child Symptom Inventory (CSI) [5, 22].

Qualified subjects were then enrolled into 1 of 2 order conditions and 1 of 3 treatment arms: cetirizine, 10 mg po qam for 2 weeks (*n* = 12); methylphenidate, 20 mg if <65 kg and 40 mg if ≥65 kg po qam for 2 weeks (*n* = 12); or cetirizine 10 mg po qam + methylphenidate, 20 mg if <65 kg and 40 mg if ≥65 kg po qam for 2 weeks (*n* = 12). All subjects crossed over to placebo for a period of 2 weeks during each treatment period.

Subjects were evaluated at baseline and at the end of each treatment arm. Assessment included allergic rhinitis endpoint measures (measuring symptoms of rhinoconjunctivitis such as sneezing, runny nose, itchy eyes, watery eyes, and stuffy nose on a scale of 0–4); the Total Symptom Severity Complex (TSSC); and the Rhinoconjunctivitis Quality of Life Questionnaire (RQLQ) [23, 24] for allergy and the Child Behavior Checklist (CBCL) [25], Conners' Rating Scale-Revised (CRS-R) [26], Conners' Continuous Performance Task (CPT) [27], and the California Verbal Learning Test for ADHD symptoms [28].

NGF levels in serum were measured using a 2-site immune-enzymatic assay (ELISA). NFG was detected in sandwich ELISAs according to the manufacturer's instructions (R&D Systems, Minneapolis, MN, USA). All assays were performed on F-bottom 96-well plates (Nunc, Wiesbaden, Germany). Tertiary antibodies were conjugated to horseradish peroxidase. Wells were developed with tetramethylbenzidine and measured at 450 nm. Neurotrophin content was quantified against a standard curve.

## 3. Statistical Analysis

Baseline scores and demographics for each of the 3 treatment groups were compared using MANOVA tests, which are best suited to analyses involving several variables. Within each group, mean scores for outcome variables were calculated for active treatment and placebo interventions. Paired *t*-tests were used to evaluate differences between treatment and placebo. Differences in change scores across the 3 treatment groups were compared (MANOVA). Additional factors, including gender, age, methylphenidate dose, depression (CDI), and anxiety (MASC), were assessed to determine any influence they may have had on scoring changes.

## 4. Results

Thirty-eight individuals (9 girls, 29 boys, aged 8 to 18 years) completed the study. This represented the normal gender distribution seen in ADHD patients. Endpoints measured allergy and ADHD-related events.

## 5. Allergic Rhinitis Endpoints

### 5.1. TSSC

**Figure 1:**
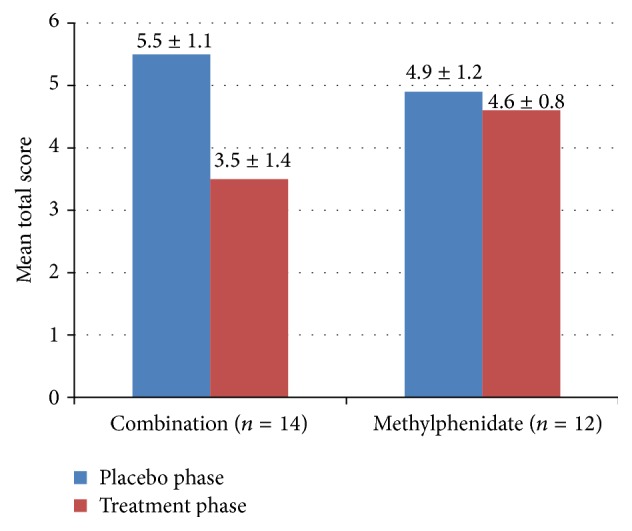
TSSC score in subjects treated with methylphenidate/cetirizine, methylphenidate, or cetirizine compared to placebo.

For seasonal allergic-related symptoms, the 2-drug combination proved to be superior to either drug alone in reducing the TSSC (Figure 1). Scores were reduced for the drug combination and for methylphenidate; there was no significant reduction for cetirizine. Rhinoconjunctivitis symptoms scores followed the same trend.

### 5.2. RQLQ

When compared to cetirizine, methylphenidate had a superior effect on general scores and showed greater reduction of general symptoms (methylphenidate: 8.1 to 5.2 points; cetirizine: no improvement). The drug combination decreased general symptoms from 9.1 to 7.2.

The same phenomena were observed in nasal and eye symptoms. For example, for nasal symptoms, methylphenidate reduced scores from 9.2 to 6.1 versus the combination of drugs (8.1 to 6.0). For eye symptoms, combination treatment reduced scores from 7.8 to 5.2, while methylphenidate alone demonstrated a 7.8 to 3.2 reduction.

Both methylphenidate and cetirizine displayed the same positive effect on emotional scores, showing reductions from 6.2 to 3.2 and 4.1, respectively.

## 6. ADHD Endpoints

### 6.1. Conners' Continuous Performance Task (CCPT)

Generally, when a slow reaction time occurs with elevated omission (missing) and commission (number of times the individual responded to a nontarget) errors, there is evidence of inattention.

The omission error rate increased when subjects received cetirizine and methylphenidate individually. However, the combination drug group elicited a significant decrease in the error rate compared with placebo.

Cetirizine lowered the commission rate from 27 to 18 and methylphenidate reduced it from 17 to 15. No effect was noted in the combination group.

Hit reaction time (HRT) is defined as the mean response time (in milliseconds) for all target responses over all 6 time blocks. High HRT scores reflect slow response times. The combination treatment regimen had the most significant effect on HRT scores.

### 6.2. Conners' Rating Scale-Revised (CRS-R)

The CRS-R, in the hyperactivity and ADHD index scores, was reduced from 70% (placebo group) to 62% (methylphenidate/cetirizine group). In contrast, there was no improvement in scores when the drugs were administered individually.

Regarding inattention, the combination group also displayed the most improvement, with a reduction from 69% to 59%; in the individual drug groups, the change was minor (70% to 68%). This trend continued with the oppositional scores: there was a reduction of 64% to 59% in the combination group, while the groups who received cetirizine or methylphenidate alone experienced only a 61% to 60% change, respectively.

### 6.3. ADHD *t*-Scores: Averages of All Scores

Methylphenidate and cetirizine had almost identical effects on *t*-scores, reducing the totals from 78 to 72 and 48 to 43 points, respectively. When evaluating inattention and oppositional *t*-scores, we observed the same trend; in particular, methylphenidate and cetirizine produced almost identical results in improving ADHD endpoints. The drug combination produced results superior to those derived from using the drugs individually.

### 6.4. NGF Assay

A large body of evidence shows that nerve growth factor (NGF) exerts biological activity on the immune system, thereby influencing allergic diseases and asthma [29–31]. In our previous studies, we noted that enhanced NGF levels were found in patients with ADHD (compared to those in non-ADHD patients) [32, 33].

**Figure 2:**
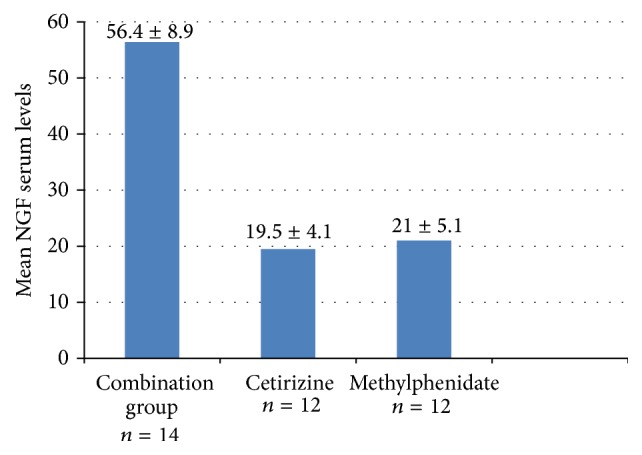
Serum NGF values in subjects treated with methylphenidate/cetirizine, methylphenidate, or cetirizine compared to placebo.

When NGF serum levels were measured in both the treatment and placebo phases of this study, a significant reduction was noted in the treatment group versus the placebo group. In the methylphenidate group, the reduction in serum NGF levels was 21 ± 5.1 ng/mL; in the cetirizine group, it was 19.5 ± 4.1 ng/mL. In the combined treatment, the greatest reduction was noted at 56.4 ± 8.9 ng/mL (Figure 2).

Overall, for the majority of measures for allergic rhinitis, ADHD, and NGF analysis, the methylphenidate and cetirizine combination produced a greater impact than did either drug individually or placebo.

## 7. Discussion

A number of studies have suggested a link between allergies and ADHD [34–38]. Two of these studies found a link between food allergies and neuropsychiatric conditions such as depression, anxiety, and ADHD [34, 38], while others looked at immune dysregulation [33], asthma medications [37], and atopic eczema [35]. Our study supports the hypothesis that, in children diagnosed with ADHD, there is a subset of children who also suffer from atopic disease. We propose that a cascade of events initiated by various stimulants (i.e., environmental factors, food, stress, or infection, in a person who is genetically predisposed to atopy) may trigger an immune inflammatory cascade that may lead to the neuroimmune inflammation presented clinically as ADHD.

In this study, we demonstrated an improvement in ADHD scores by using drugs known to regulate allergic inflammation (e.g., antihistamines such as cetirizine); the benefit of stimulant medication on allergic inflammation (e.g., reduction in TSSC scores by methylphenidate); and the synergistic effect of methylphenidate and cetirizine on both allergic and neurological symptoms.

Based on these results, we suggest that there is some overlap in the mechanism of action of allergy and ADHD in patients with a comorbid diagnosis. Moreover, by using a synergistic approach, the individual effect of each drug on allergic or neurological endpoints is enhanced.

This study also raised questions and concerns for which there are currently no obvious answers. For example, why did not we get the expected results in reduction of TSSC with antihistamines in the comorbid population? Why did stimulant drugs have the same beneficial effect as antihistamines in reducing TSSC scores? And lastly, why did the combination drugs have a superior effect?

In regard to the first point, patients diagnosed with ADHD have an unfavorable perception of life and, thus, antihistamine therapy may not be sufficient to control their TSSC scores. Additionally, in patients with specific comorbid disease, different cetirizine dosages may be required to achieve full control of allergy symptoms.

Regarding the second question, over the years we have observed that stimulants, specifically methylphenidate, have an antihistamine effect (measured by suppressing wheal and flare). This antihistaminic effect may explain some of the improvements in TSSC scores in the comorbid population.

In response to the third question, antihistamines, specifically cetirizine, may have an anti-inflammatory effect [39, 40]. This regulation of inflammatory processes can positively impact ADHD endpoints in children with comorbid diseases. By adding to the antihistamine effect of cetirizine and the stimulant effect of methylphenidate, a synergistic effect may have been induced on both allergy and ADHD scores.

The linkage between allergic and mental disorders may have a bidirectional mechanism: allergic triggers induce either a neuroimmune inflammation with a direct effect on the CNS or CNS stimuli (e.g., stress, anxiety, or other factors release neurostimulants that impact allergic inflammation) [41]. As we have seen, allergic disorders can lead to significant cognitive distortions, especially during the peak allergy season (e.g., ragweed season); interestingly, this effect was found only in a subgroup of patients [8]. These disorders have also been associated with such conditions as depression, anxiety, and panic disorder, thus raising the question as to whether these shared maladies are the result of shared genetic risks or some common mechanism, which may exist between allergic disorders and mental diseases [42].

The mechanisms by which NGF may be involved in allergic disease or in comorbid disease of allergy and ADHD are unknown, but a small number of theories have been postulated. One theory is that, upon mast activation and differentiation, NGF release is increased. The differentiation of human mast cell subtypes that could perpetuate human allergic reactions is dependent on NGF. NGF is increased in biologic fluids of allergy/immune-related diseases and has been evaluated as a TH2 cytokine with a modulator role in allergic inflammation and tissue remodeling. In patients with allergic bronchial asthma, for example, elevated levels of neurotrophins have been reported. In a time course study of bronchoalveolar lavage fluid, levels of NGF correlated with the inflammatory response. It was shown that NGF, specifically, enhances inflammation in allergic early-phase response [43].

The role of NGF in allergic-related diseases is unclear [44]. Another possibility is that, upon allergic response with a matching genetic underline, release of neurotrophins can induce brain inflammation.

As part of neuroimmune healing, we need an intact apoptosis process. NGF belongs to the super family of CD40, which plays a crucial role in this process. Any failure in NGF signaling or regulation may lead to an abnormal apoptosis process and, as a result, may cause persistent inflammation. In turn, this persistent inflammation may be the cause of neurological manifestation of ADHD.

Our findings support the hypothesis that (1) atopy may, in some cases, contribute to the pathogenesis of ADHD; (2) NGF plays a role in the pathogenesis of comorbid diseases; and (3) neurological triggers (e.g., anxiety, stress, or environmental factors, including food) may trigger the comorbid condition.

In this study, the role of NGF was demonstrated by the modulating effect of the antihistamines and stimulants on NGF levels in patients with the comorbid diseases. NGF suppression had a direct correlation to clinical improvement.

In conclusion, our research clearly demonstrated that the combination of an antihistamine (cetirizine) and a stimulant (methylphenidate) produced superior results when treating children with comorbid allergic rhinitis and ADHD, enhancing the efficacy of treatment for both allergic and ADHD symptoms. Our findings also suggest that (1) in patients with comorbid disease the treatment approach should include antihistamines and (2) a synergistic effect may be achieved by using both drugs, thus making it possible to reduce the dosage of the stimulant. We also discovered that NGF may play an integral role in these processes. NGF measurements followed the trends of allergic symptoms and ADHD and could possibly be used as a marker for measuring the severity of either condition. Further studies in larger populations are needed.
